# Different Serotonergic Expression in Nevomelanocytic Tumors 

**DOI:** 10.3390/cancers2021166

**Published:** 2010-06-07

**Authors:** Clara Naimi-Akbar, Markus Ritter, Sasika Demel, Husameldin El-Nour, Mari-Anne Hedblad, Efrain C. Azmitia, Klas Nordlind

**Affiliations:** 1 Dermatology and Venereology Unit, Department of Medicine, Solna, Karolinska Institutet, Karolinska University Hospital, Solna, Sweden; E-Mails: Clara@linkdata.se (C.N.-A.); Markus.Ritter@gmx.net (M.R.); Sasika@demel.se (S.D.); Husameldin.Elnour@ki.se (H.E.-N.); Mari-Anne.Hedblad@karolinska.se (M.-A.H.); 2 Department of Biology and Psychiatry, New York University, NY, USA; E-Mail: eca1@nyu.edu

**Keywords:** melanoma, serotonin, receptor, serotonin transporter protein, immunohistochemistry

## Abstract

The neuromediator serotonin (5-hydroxytryptamine; 5-HT) has been proposed to play a role in tumor progression. Thus, the aim of the present investigation was to determine whether alterations in the serotonergic system occur in nevomelanocytic tumors. For this purpose, paraffin-embedded biopsies of superficial spreading malignant melanoma (SSM), dysplastic compound nevi (DN) and benign compound nevi (BCN) were characterized with regard to their expression of 5-HT, the 5-HT1A and 5-HT2A receptors, and the serotonin transporter protein (SERT), by immunohistochemical analysis. Melanocytes in the region surrounding the tumor were found to express both the 5-HT1A and 5-HT2A receptors. Tumor cells that immunostained positively for the different serotonergic markers were observed in the suprabasal epidermis of DN tissue and, to an even greater extent, in the case of SSM. Furthermore, some of these latter cells expressed both 5-HT1AR and 5-HT2AR. The level of expression of 5-HT1AR at the junctional area was lower for SSM than for DN or BCN. As the degree of atypia increased, the intensity of tumor cell staining in the dermis for 5-HT1AR and SERT declined. Vessel immunoreactivity for 5-HT2A was more intense in SSM than in BCN tissue. Round-to-dendritic cells that expressed both SERT and 5-HT1AR were seen to infiltrate into the dermal region of the tumor, this infiltration being more evident in the case of DN and SSM. These latter cells were also tryptase-positive, indicating that they are mast cells. Thus, alterations in serotonergic system may be involved in nevomelanocytic tumors and mast cells may play an important role in this connection.

## 1. Introduction

Progression of a tumor to cutaneous malignant melanoma begins with the loss of cell cycle control and dysregulated proliferation of intraepidermal melanocytes. Subsequent alterations allow the tumor cells to invade and migrate through the dermis, thereby gaining access to the vascular spaces. In addition, some of these cells are also found in the suprabasal region of the epidermis. 

Serotonin (5-hydroxytryptamine; 5-HT) participates in the regulation of a wide variety of physiological processes [1], including basic cell functions such as proliferation, differentia-tion/maturation, migration and maintenance of vessel tonus [2]. The serotonin transporter protein (SERT) regulates the concentrations of 5-HT in the synaptic cleft by mediating re-uptake of secreted 5-HT, thereby terminating its action. Of the 21 different receptor (R) subtypes to 5-HT that have been cloned in mammals to date [1,3], the 5-HT1AR is one of the most extensively characterized. This receptor is present both presynaptically, as an autoreceptor on raphe cells, located on the midline of the brainstem, and postsynaptically on target neurons located in the hippocampus. The 5-HT1AR has been proposed to promote cell differentiation, while the opposite effect has been reported for the 5-HT2 receptor family [2].

Via various receptors 5-HT is thought to function as a growth factor for different malignancies *in situ* and/or for associated cell lines , e.g., pulmonary carcinoma, prostate carcinoma, pancreatic carcinoid, bladder cancer, glioma and breast cancer [4,5,6], while SERT influences the development of colorectal tumors [7] and breast cancer [8,9]. 

The cells present in human skin, including melanocytes and even melanoma cells, express the enzymatic pathways responsible for 5-HT biosynthesis [10,11,12,13,14]. 5-HT receptor gene expression has been documented in various skin cells including melanocytes and melanoma cells, and 5-HT can affect skin cell proliferation depending on the growth conditions [15,16]. In this respect an inhibitor of 5-HT uptake, 6-nitroquipazine, was reported to inhibit melanization in human melanoma MM96E cells [17], while in the human melanoma cell line SK-MEL-188, 5-HT itself inhibited DMEM-induced melanogenesis [18]. 

Here, we have examined the expression of 5-HT, and its 5-HT1A and 5-HT2A receptors and SERT, in nevomelanocytic tumors. For this purpose, benign compound nevi (BCN), dysplastic compound nevi (DN) and superficial spreading malignant melanoma (SSM) were subjected to immunohistochemical analysis employing a technique involving biotinylated-streptavidine. We also performed double staining for tryptase- a mast cell specific protease, which is involved in the innate and adaptive immunity, as well as in malignant melanoma, where it plays an important role in neovascularization [41]. 

## 2. Materials and methods

### 2.1. Tissue Specimens

Paraffin-embedded biopsies of BCN, DN and SSM (Clark levels II-IV) were examined. The SSM biopsies were from the extremities of five females, with a mean age of 70 (range 58-90) years, and from the trunk and arm in four males, with a mean age of 56 (range 38–74 years). The DN nevi were from the extremities of two females, 41 and 48 years, and from the trunk and thigh of seven males, with a mean age of 41 (range 27–70) years. The BCN biopsies were from the face and trunk of four females, with a mean age of 29 (range 21–37) years and from the face, trunk and thigh of four males, mean age 18 (range 13–26) years.

The DN were defined as nevi showing besides intraepidermal lentiginous hyperplasia of melanocytes also extension of the junctional component beyond the intradermal component, bridging of the rete ridges, lamellar or concentric fibrosis, a stromal response of inflammatory cells and random cytological atypia of the cells. 

The study was approved by the local ethical committee at the Karolinska University Hospital. 

### 2.2. Immunohistochemical Procedure

Sections 4 µm thick were deparaffinized using Bio-Clear (Bio-Optica, Milano, Italy) for 10–15 min, followed by sequential rehydration with progressively decreasing concentrations of ethanol in water (100, 95 and 70%, 2 min in each). These sections were then incubated with polyclonal antibodies against 5-HT (20080, dilution 1:10,000; Diasorin, Stillwater, MN, USA) or 5HT1AR, or monoclonal antibodies directed towards 5-HT2AR (diluted 1:150) and SERT (ST51-1; 1:20,000; MAB Technologies, Stone Mountain, GA, USA). The SERT antibody was raised against a peptide (amino acids 51–66) coupled to KLH by addition of an N-terminal cysteine. The polyclonal antibody towards rat 5-HT1AR (SlA-170) was raised in rabbit and used at a dilution of 1:1,000. This antibody is directed against amino acid residues 170–186 in the second extracellular loop of this receptor [19], a sequence that exhibits complete homology between humans and rats. This antiserum labels a major 64-KDa protein in immunoblots of proteins solubilized from neonatal rat brain and from different recombinant systems, in particular cells transfected with constructs carrying the coding sequence for 5-HT1AR [20]. The mouse monoclonal antibody against the 5-HT2AR (G186-1117) employed was a generous gift from Pharmingen (San Diego, CA, USA). It was directed against amino acid residues 1‑76 in the *N*-terminal extracellular domain of the human 5-HT2AR [21]. This antibody has been shown to stain reactive, but not inactivated human astrocytes [22], Purkinje cell bodies and dendrites [23]. Incubation with the primary poly- or monoclonal antibodies was followed by incubation with a secondary biotin-labelled goat anti-rabbit antibody (BA-1000) or horse anti-mouse antibody (BA-2000), both in dilution 1:200 and from Vector, Burlingame, CA, USA, followed by visualization by treatment with fluorochrome Cy2-labeled streptavidine (PA42001, 1:2,000; Amersham Pharmacia Biotech, Uppsala, Sweden). All antibody solutions were diluted in phosphate-buffered saline (PBS), pH 7.4, containing 1% bovine serum albumine (BSA) (A9418, Sigma Aldrich, Stockholm). 

As a control, the antibody preparation directed towards 5-HT1AR was preadsorbed with the corresponding antigenic peptide (see above) (0.1 mg/mL, synthesized by Ross-Petersen AS, Horsholm, Denmark) and the antibody against 5-HT, with 5-HT (85030, 10^−5^ mol/L; Sigma-Aldrich). These preadsorptions omitted the staining. Moreover, control incubations with unspecific mouse IgG of the same isotype (X0931, Dako, Glostrup, Denmark) and at the same dilution as the monoclonal antibodies, as well as incubations from which the primary antibodies were omitted, produced no immunostaining.

### 2.3. Double Staining

The SSM tissue specimens were double-stained for the 5-HT1A and 5-HT2A receptors. In this connection, following incubation with 10% normal horse serum for 15 min at room temperature and thereafter with the monoclonal antibody against the 5-HT2AR overnight at 4 ^o^C, the sections were exposed to the biotinylated horse anti-mouse IgG and thereafter to streptavidine-conjugated Texas Red (SA-5006, dilution 1:2,000; Vector). Subsequently, after incubation with 10% normal swine serum for 15 min at room temperature, the sections were treated overnight at 4 ^o^C with the polyclonal antiserum against 5-HT1AR, followed by fluorescein isothiocyanate (FITC)-conjugated swine anti-rabbit IgG (F0205, dilution 1:30; Dako), as the secondary antibody.

In order to identify infiltrating inflammatory, non-tumoral, cells in the tumor region of SSM, double-staining for 5-HT1AR and SERT was also performed. For this purpose, the sections were incubated sequentially with the polyclonal antibody against the 5-HT1AR, a biotinylated goat anti-rabbit antibody, and streptavidine-conjugated Texas Red. The sections were then incubated with the monoclonal antibody against tryptase (MAB1222, 1:5,000; Chemicon, Temecula, CA, USA) and FITC-conjugated donkey anti-mouse antibody (F0261, dilution 1: 150; Jackson ImmunoResearch, West Grove, Pennsylvania, USA) under the same conditions as described above. A similar procedure was employed to stain for SERT, except that in this case a polyclonal rabbit antibody to tryptase (diluted 1:1,000; a kind gift from from Prof. I. Harvima, Dept. of Dermatology, University of Tampere, Finland) and the FITC-conjugated swine anti-rabbit antibody was utilized.

In the final step, all sections were mounted with glycerol/PBS (10:1) containing 0.1% paraphenylenediamine and a coverslip applied.

### 2.4. Microscopy

The epifluorescence of the sections was examined using a Nikon Microphot-FX microscope. FITC/Cy2 fluorescence was monitored with a Nikon B-2A filter cube (excitation at 450–490 nm with an emission filter allowing wavelengths of 520–560 nm to pass) and Texas Red fluorescence with a Nikon G-1B filter cube (excitation at 546 ± 5 nm with a barrier filter at 590 nm). Double-staining was examined utilizing a Nikon/Rhodamine double-filter. The sections were photographed with a Nikon digital camera (DXM 1200) attached to the fluorescent microscope and the images transferred to a computer. 

All samples were evaluated independently by two observers (CN-A and KN). The degree of occurrence of cells in the suprabasal region of the epidermis, expression at the junction between epidermis and dermis, the signal strength of the dermal tumor cells, vessel immunoreactivity and infiltration of SERT- and 5-HT1AR-positive inflammatory non-tumor cells into the tumoros dermal area were all scored as 0 (none), 1 (slight), 2 (moderate) or 3 (intense).

### 2.5. Statistics

Multiple comparisons of the three types (BCN, DN and SSM) were performed by use of analysis of variance, ANOVA. In the case of a statistically significant result in the ANOVA, statistical comparisons between two arbitrary groups were made by use of the post-hoc test proposed by Fisher to control for multiplicity [24,25]. In order to evaluate hypotheses of variables in contingency tables, the chi-square test was used. In addition to that, descriptive statistics were used to characterize the data and the mean and standard deviation has been given. All analyses were carried out by use of statistical software (the SAS system for Windows 9.1. SAS Institute Inc., Cary, NC, USA). In the case of a statistically significant result the probability value (P-value) has been given.

## 3. Results

### 3.1. General Findings

No specific labeling occurred following pre-adsorption of the antibodies against 5-HT and 5-HT1AR, or when isotypic monoclonal antibodies were utilized instead of monoclonal antibodies towards 5-HT2AR and SERT (data not shown).

### 3.2. Migration of Cells into the Suprabasal Epidermis

**Figure 1:**
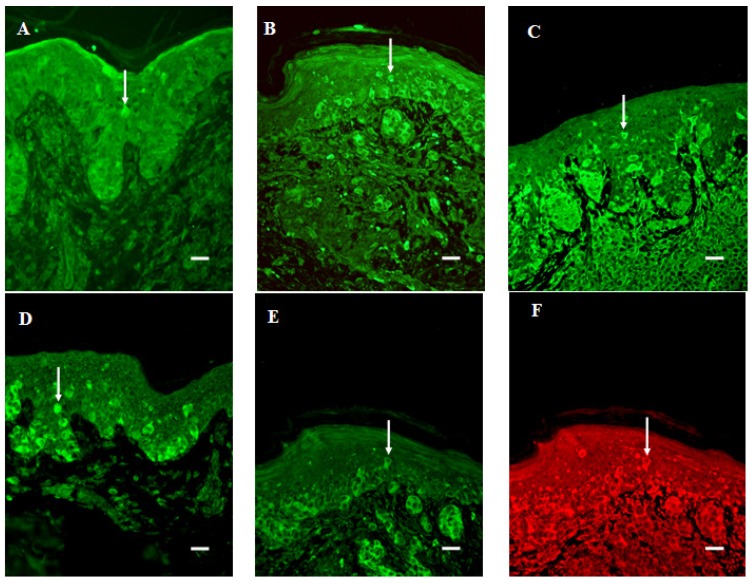
Melanoma cells in the suprabasal epidermis of SSM tissue that immunostain positively for 5-HT (arrow in A), 5-HT1AR (arrow in B), 5-HT2AR (arrow in C), or SERT (arrow in D). (B) Decreased junctional expression of 5-HT1AR in this same tissue. Double staining for 5-HT1AR (arrow in E, FITC) and 5-HT2AR (arrow in F, Texas Red). Bar is 20 μm.

Melanocytes present in regions surrounding the tumor were found to express both the 5-HT1A and 5-HT2A receptors. The frequency of 5-HT positive cells in the suprabasal epidermis increased in the order BCN < DN < SSM (P < 0.05). In addition, the expression of the 5-HT1A and 2A receptors in this region was lower in BCN than in SSM, and DN, tissue (P < 0.05 in each case). Moreover, staining for SERT was more intense in SSM than in BCN and DN tissue (*P* < 0.05 in each case) (Table 1; Figures 1A–D). Some of these cells double-stained positively for both 5-HT1AR and 5-HT2AR (Figures 1E, F).

### 3.3. Expression in the Junction Area between Epidermis and Dermis

The level of expression of 5-HT1AR in this junctional region was significantly higher in BCN than in SSM (P < 0.05) and in DN than in SSM, tissue (P < 0.05) (Figure 1B). In contrast the junctional expression of 5-HT, SERT and 5-HT2AR did not change with increasing atypia.

### 3.4. The Strength of the Signal from Tumor Cells in the Dermis

The intensity of immunostaining of tumor cells in the dermis for 5-HT was rather similar in the different tumors. However, the immunosignal for 5-HT1AR, as well as for SERT, was higher in BCN than in either DN or SSM tissue (P < 0.05 in both cases). 

### 3.5. Vessel Immunoreactivity

**Figure 2:**
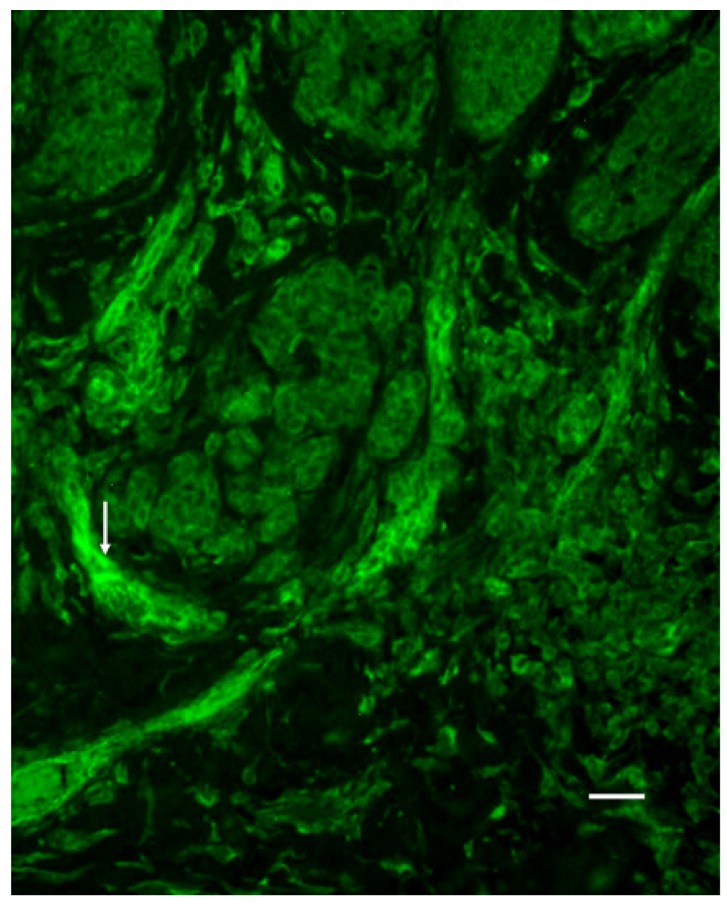
Immunostaining for 5-HT2AR in the vessels (arrow) of SSM tissue. Bar is 20 μm.

The immunoreactivity of the vessel walls for 5-HT2AR was higher in SSM than in BCN tissue (P < 0.01; Figure 2). 

**Table 1:** Expression of 5-HT, SERT, 5-HT1AR and 5-HT2AR in BCN, DN and SSM.

		Cells in the suprabasal *epidermis*	*Junction area*	*Dermis* Signal strength in tumour cells	Vessels	i SERT	i 5-HT1A
BCN	5-HT	0	1.0 ± 0.7	1.0 ± 0	0 ± 0		
	SERT	0.3 ± 0.4	1.0 ± 0.7	1.8 ± 0.4	0 ± 0	0.1 ± 0.3	
	5-HT1AR	0	2.6 ± 0.5	1.9 ± 0.3	0 ± 0		0 ± 0
	5-HT2AR	0.6 ± 0.7	0.9 ± 1.3	1.9 ± 0.6	0.8 ± 1.2		
DN	5-HT	0.6 ± 0.7	0.8 ± 0.8	1.1 ± 0.3	0.1 ±0.3		
	SERT	0.7 ± 0.5	1.7 ± 0.7	1.1 ± 0.6	0 ± 0	1.5 ± 0.5	
	5-HT1AR	1.5 ± 1.1	2.0 ± 0.9	1.4 ± 0.5	0.4 ± 0.7		0.6 ± 0.5
	5-HT2AR	1.8 ± 0.4	1.9 ± 0.9	1.7 ± 0.5	1.6 ± 1.3		
SSM	5-HT	1.5 ± 0.5	0.5 ± 0.5	1.3 ± 0.6	0.2 ± 0.4		
	SERT	1.8 ± 0.6	1.4 ± 0.7	1.2 ± 0.4	0 ± 0	0.9 ± 0.7	
	5-HT1AR	1.9 ± 0.9	1.1 ± 0.6	1.3 ± 0.4	0.4 ± 0.5		1.1 ± 0.6
	5-HT2AR	2.1 ± 0.6	2.0 ± 1.0	1.6 ± 0.5	2.1 ± 0.6		

i = infiltration in the tumour area of immunoreactive inflammatory cells.

### 3.6. Infiltration of SERT- and 5-HT1AR-Immunoreactive Cells into the Dermal Region of the Tumor

**Figure 3:**
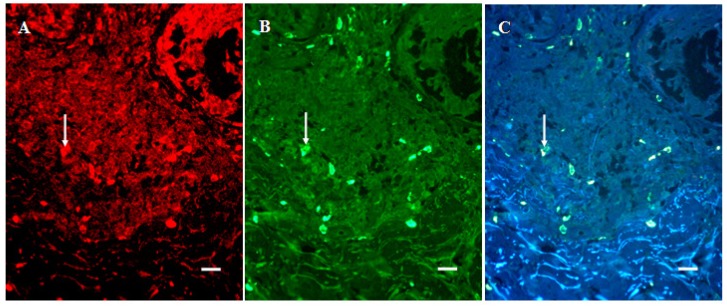
Double-staining of SERT- positive inflammatory cells present in the tumorous dermal region of SSM tissue. (A) SERT- positive cells (arrow, Texas Red). (B) tryptase-positive cells (arrow, FITC). (C) SERT and tryptase (arrow). Bar is 20 μm.

Round- to- dendritic cells in the dermal region of the tumor area immunostained positively for SERT and 5-HT1AR. In fact, these SERT- positive cells exhibited the most intense cytoplasmic signal of all of the structures that stained positively for SERT. The number of cells in this area that expressed SERT was higher (P < 0.05) in both DN and SSM than in BCN tissue, being highest in the case of DN. Double-staining revealed that these SERT- positive non-tumor cells also expressed tryptase (Figure 3). 

**Figure 4:**
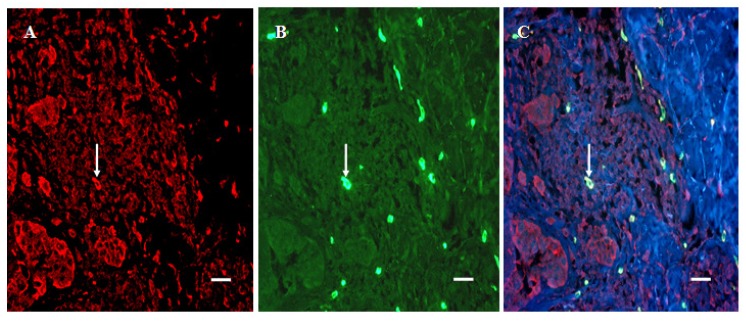
Double-staining of 5-HT1AR-positive inflammatory cells in the tumorous dermal region of SSM. (A) 5-HT1AR-positive cells (arrow,Texas Red). (B) tryptase-positive cells (arrow, FITC). (C) 5-HT1AR and tryptase (arrow). Bar is 20 μm.

Moreover, the number of non-tumoral cells that stained for 5-HT1AR was greater in SSM, than in DN (P < 0.05) or BCN tissue (P < 0.001). Here, as well, double-staining revealed that these same cells were tryptase-positive (Figure 4).

## 4. Discussion

In the present investigation, cells that immunostained positively for all of the serotonergic markers examined—*i.e.*, 5-HT, 5-HT1AR and/or 5HT2AR, and/or SERT—were detected in the suprabasal epidermis of nevomelanocytic tumors. Furthermore, the level of 5-HT1AR expression at the junction between tumorous and non-tumorous tissue decreased with an increasing degree of atypia. In addition, expression of both 5-HT1AR and SERT by tumor cells in the dermis was lower in DN and SSM than in BCN tissue. 

This attenuation of the expression of the ligand, as well as of its receptors and transporter protein in the dermal region as the degree of the anaplasia increased might reflect poor differentiation of the melanoma cells. Indeed, as reported in studies on neural cells [2], 5-HT action via its receptors may promote a differentiation. 

Certain of the cells in the epidermis expressed both the 5-HT1A and 5-HT2A receptors. Such colocalization of these receptors has also been observed in different regions of the brain [26,27,28], suggesting that 5-HT may exert opposing effects on one-and-the same neuron. 5-HT2AR may interact with 5-HT1AR, to desensitize the latter [29]. 

5-HT is capable of inducing the formation of DNA adducts, DNA strand breaks and chromosomal aberrations, as well as of inhibiting DNA synthesis [30]. This substance can also induce apoptosis in cultured neuronal cells [31] and promote the death of Burkitt lymphoma cells, via a mechanism involving 5-HT uptake [32]. Later, these same authors reported that SSRIs induce apoptosis in these lymphoma cells [33]. 

Moreover, paroxetine and fluoxetine cause apoptosis in glial and neuroblastoma cell lines [34]. On the other hand, Brandes and co-workers [35] found that antidepressants (including fluoxetine) stimulate the growth of melanoma cells that are injected into mice. An impact of SSRIs on the risk of developing breast cancer [8,9] and colorectal tumors [7] has earlier been discussed. While there might be an increased risk for breast cancer associated with the use of SSRIs, a decreased risk of colorectal cancer was correlated with a high SSRI dose It has been proposed that SERT is a novel target for the anti‑tumor activity of amphetamine analogs; whereas the impact of antidepressants on malignant B cells might be independent of the transporter itself [36].

In addition to direct effects of the ligand on cells, involving its receptors and SERT, serotonergic effects on interstitial collagenases, mediated by the 5-HT2AR subtype, might occur [37]. Thus, an antagonist of this receptor reduces the expression of matrix metalloproteinase [38].

The vessels in SMM tissue demonstrate elevated immunoreactivity for the 5-HT2AR. The vasoconstrictive response of arterioles that supply tumors to 5-HT is more pronounced than in the case of other vessels [39]. Although vasoconstriction is usually mediated by 5-HT2 receptors, the 5-HT1AR-positive arterioles that penetrate into tumors exhibit a specific type of vasoconstriction [40].

SERT- and 5-HT1AR- positive mast cells were found here to infiltrate the tumor tissue to an ever greater extent as the degree of atypia increases. Extensive tumor vascularity and the presence of numerous tryptase-positive mast cells have been correlated to poor prognosis in patients suffering from melanoma. Mast cells are known to release a variety of factors that enhance angiogenesis. For example, fibroblast growth factor-2 secreted by the tumor cells and tryptase secreted by host mast cells have been reported to act together to promote angiogenesis [41]. In addition, this tryptase is capable of activating matrix metalloproteinases [42], and both tryptase itself as well as these activated proteinases can degrade various components of the pericellular/extracellular matrix. At the same time, the mast cells may be part of an immune response designed to eliminate the tumor cells [43].

It has earlier been shown that melanocytes and melanoma cells, express the enzymatic pathways responsible for 5-HT biosynthesis [10,11,12,13,14] and 5-HT receptor gene expression has been documented in these cells [15,16]. The present immunohistochemical findings document alterations in the serotonergic system in nevomelanocytic tumors, alterations which might provide useful therapeutic targets for the treatment of dysplastic nevi/malignant melanoma. This might indicate the usefulness of 5-HT1A agonists and 5-HT2A antagonists or modulators of SERT in the treatment of malignant melanoma. Moreover, mast cells may play an important role in this connection.

## 5. Conclusion

Alterations in serotonergic system seem to be involved in nevomelanocytic tumors, and mast cells are expected to play an important role in this connection.
